# Polarized Light Imaging of the Myoarchitecture in Tetralogy of Fallot in the Perinatal Period

**DOI:** 10.3389/fped.2020.503054

**Published:** 2020-09-22

**Authors:** Ba Luu Truong, Pierre-Simon Jouk, Johanne Auriau, Gabrielle Michalowicz, Yves Usson

**Affiliations:** ^1^Centre National de la Recherche Scientifique (CNRS), Techniques de l'Ingénierie Médicale et de la Complexité - Informatique, Mathématiques, Applications, Grenoble (TIMC-IMAG), Grenoble, France; ^2^Department of Pediatric Cardiac Surgery, Necker Sick Children Hospital, Paris, France; ^3^Department of Pediatric Cardiology, Nhi Dong 2 Children Hospital, Ho Chi Minh City, Vietnam; ^4^Department of Genetics and Procreation, Grenoble-Alpes University Hospital, Grenoble, France; ^5^Department of Cardiology, Grenoble-Alpes University Hospital, Grenoble, France

**Keywords:** polarized light imaging, 3D architecture myocardial cells, tetralogy fallot, ventriculo infundibular fold, outlet septum

## Abstract

**Background:** The pathognomonic feature of tetralogy of Fallot (ToF) is the antero-cephalad deviation of the outlet septum in combination with an abnormal arrangement of the septoparietal trabeculations.

**Aims:** The aim of this article was to study perinatal hearts using Polarized Light Imaging (PLI) in order to investigate the deep alignment of cardiomyocytes that bond the different components of the ventricular outflow tracts both together and to the rest of the ventricular mass, thus furthering the classic description of ToF.

**Methods and Materials:** 10 perinatal hearts with ToF and 10 perinatal hearts with no detectable cardiac anomalies (control) were studied using PLI. The orientation of the myocardial cells was extracted and studied at high resolution. Virtual dissections in multiple section planes were used to explore each ventricular structure.

**Results and Conclusions:** Contrary to the specimens of the control group, for all ToF specimens studied, the deep latitudinal alignment of the cardiomyocytes bonds together the left part of the Outlet septum (OS) S to the anterior wall of the left ventricle. In addition, the right end of the muscular OS bonds directly on the right ventricular wall (RVW) superior to the attachment of the ventriculo infundibular fold (VIF). Thus, the OS is a bridge between the lateral RVW and the anterior left ventricular wall. The VIF, RVW, and OS define an “inverted U” that roofs the cone between the interventricular communication and the overriding aorta. The opening angle and the length of the branches of this “inverted U” depend however on three components: the size of the OS, the size of the VIF, and the distance between the points of insertion of the OS and VIF into the RVW. The variation of these three components accounts for a significant part of the diversity observed in the anatomical presentations of ToF in the perinatal period.

## Introduction

Tetralogy of Fallot (ToF) is a common cyanotic congenital heart disease. The malformation was described in 1888 by Etienne-Louis Arthur Fallot as the association of inter-ventricular communication, sub-pulmonary stenosis, overriding of the aorta, and hypertrophy of the right ventricle. These anatomical features had been described before him, but his article simply and clearly stated the clinico-pathological correlations of what he called the “maladie bleue.”

Since Fallot's publication, knowledge about this malformation has increased and the pathognomonic feature of ToF is now defined as the antero-cephalad deviation of the outlet septum in combination with an abnormal arrangement of the septoparietal trabeculations (1–4).

A keyword search of PubMed for “Tetralogy of Fallot and Anatomy” produces many articles, however only 54 articles focus on the cardiomyocytes of the ventricular mass and of them only six consider the 3D myoarchitecture. This reflects the difficulty of investigating the deep myocardial anatomy. Until the 21th century, anatomical studies of ToF were principally based on the description of the endocardial reliefs completed by a few destructive anatomical peelings and dissections. However, these fail to provide reliable information about the myocardial architecture (5–11).

Information about the deep myoarchitecture has now become accessible due to progress in 3D medical imaging, in particular a magnetic resonance imaging (MRI) technique called diffusion tensor imaging (DTI), Computer Tomography (CT), microCT, and Polarized Light Imaging (PLI). These techniques enable the main direction of the myocardial cells to be obtained (the first eigenvector) (12), with DTI-MRI more suitable for infant and adult hearts, while PLI can be used for perinatal hearts (13–21).

Our previous studies focused on the 3D myoarchitecture of perinatal and infant hearts with no detectable cardiac anomalies. Indeed, we showed that post-natal remodeling generates inhomogeneity in the 3D myoarchitecture of the ventricular mass and that this post-natal remodeling event is correlated with the post-natal appearance of the rotational mechanics of the left ventricle (22, 23). The present study focuses on ToF–a frequent cardiac malformation -during the perinatal period. The study of this perinatal period is important as it covers the transitional step from an embryological pattern to an infant pattern with modification by post-natal remodeling. However, though the hospital collection includes 10 perinatal hearts with ToF, it does not include any infant hearts with ToF, thanks to the reduction in the mortality of infants with ToF. It was therefore not possible in this present study to evaluate the post-natal remodeling of ToF. Hence, the objective of this article was to describe the deep 3D myoarchitecture of ToF in the perinatal period and to study the deep alignment of cardiomyocytes that bond the different components of the ventricular outflow tracts both together and to the rest of the ventricular mass.

## Materials and Methods

### Ethics Statement

Grenoble-Alpes University Hospital owns a legally declared collection of embedded tissue sections collected after autopsy for perinatal and infant death performed for a diagnostic purpose. Written consent was obtained from the parents or guardians at the time of the request for autopsy authorization and for research authorization on normal and abnormal development. The research protocol for this study was approved by the institutional review board of the Grenoble-Alpes University Hospital. Samples dedicated to research purposes were anonymized. The study was conducted in accordance with the Declaration of Helsinki.

### Materials

Group 1: 10 perinatal human hearts with no detectable cardiac anomalies.

Group 2: 10 perinatal hearts with ToF confirmed by autopsy (four with pulmonary stenosis and six with pulmonary atresia).

### Histological Preparation and PLI

**Figure 1:**
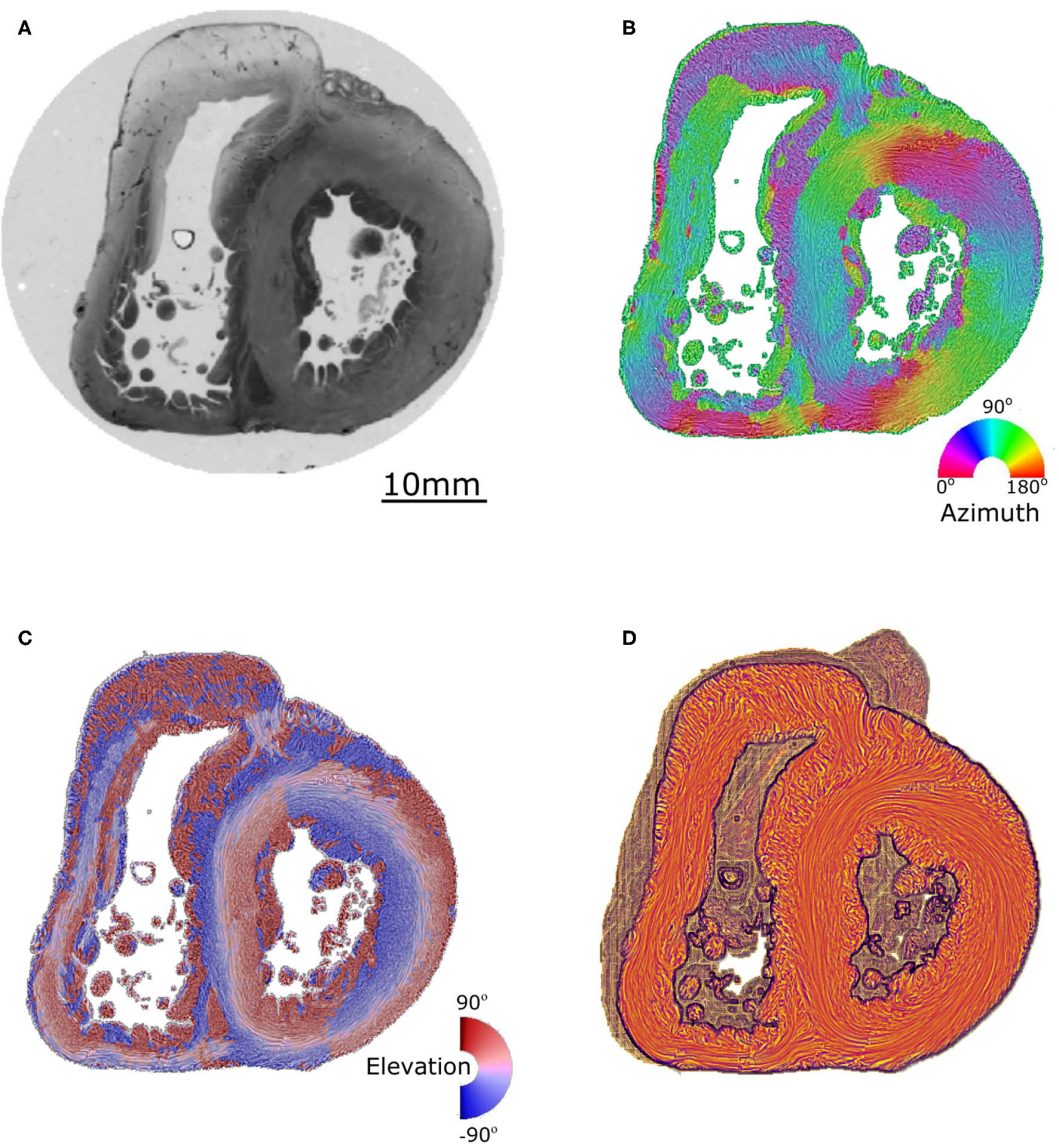
Illustration of the different maps obtained by PLI. **(A)** Short-axis section (500 μm thickness) made after resin embedding and viewed in transmitted light. **(B)** False color azimuth map of the section with superimposition of LIC texture (0–180° range). **(C)** False color elevation map with superimposition of LIC texture (−90 to +90° range). **(D)** Orientation map limited to “streamlines” representation in LIC texture. The virtual dissection plan is false-colored in orange-brown, with the surface of the basal ventricular mass in brown.

A detailed description of the protocol used can be found in previous publications (24–27) and is briefly summarized here. Heart samples were fixed in a solution of 4% neutral buffered formaldehyde. The ventricular mass was removed by cutting the atriums 1 mm above the atrioventricular groove and the great arteries 3 mm from the semi-lunar valves. The ventricular masses were then embedded in methyl methacrylate (MMA). Before sectioning, three fiducial markers were etched perpendicular to the coronal plane on the sides of the MMA resin block. A series of 500 μm thick sections were cut with a diamond wire saw (ESCILR) along the heart short axis (Figure 1A). Due to the thickness of the wire saw, 500 μm of material was lost and consequently, the section spacing was 1,000 μm. The use of MMA made it possible to cancel the form birefringence of the collagen because MMA and collagen refraction indices matched well. We previously demonstrated that in this condition the birefringence of the myocardium is essentially due to the crystalline uniaxial positive birefringence of myosin (24). The orientation information can then be extracted using classical PLI techniques on a three-axis rotation stage adapted to biology, in particular with respect to the low value of birefringence and the size of the samples. The mean orientation of all myosin filaments contained inside a voxel [100 × 100 × 500 μm] was measured. Such a voxel contains ~500 myocardial cells. The orientation information is expressed by two components: the azimuth angle (Figure 1B), which is the angle between the east–west axis of the optical bench stage and the projection of the voxel principal orientation on the stage plane (0–180° range); and the elevation angle (Figure 1C), which is the angle corresponding to the obliquity of the voxel principal orientation with respect to the plane of the section. In other words, it is the way the voxel principal orientation escapes from the section plane (−90 to +90° range). Both these angles are defined in an absolute 3D Cartesian coordinate system (Figure 1D). When dealing with 2D and 3D representations, this Cartesian system has the classical drawback of the meridian and anti-meridian discontinuities but it has the great advantage of not suffering from the reconstruction artifacts observed when cylindrical or semi-ellipsoid coordinate systems are applied to non-axisymmetric structures (24–27).

### Visualization, Virtual Dissection, and Morphometric Measurements

To provide readable and comprehensive information on myocardial cell orientations, we used a streamline representation based on a classical line integral convolution (LIC) algorithm. A texture image is built using tractographies in regions of limited size (5 voxel). This LIC texture integrates both the azimuth and elevation angles and can be read independently or overlaid on the azimuth and elevation maps. Such a streamline representation avoids the problem of the loss of bijectivity for tractographies in large regions (region >25% ventricular mass).

Virtual dissection of the ventricle volume was achieved using the ImageJ (*http://rsb.info.nih.gov/ij*) Volume Viewer plugin (Kai Uwe Barthel, Internationale Medien informatik, HTW Berlin, Berlin, Germany *http://rsb.info.nih.gov/ij/plugins/volume-viewer.html*). This made it possible to reconstruct the full ventricular mass while defining section planes with arbitrary orientation in order to investigate the inner organization of the streamline representation (LIC texture—cardiomyocytes alignment orientation).

#### Length Measurements

The apico-basal length (Abl) of each heart of the two groups was measured on a segment perpendicular to the atrioventricular junction ending at the apex of the ventricular mass.

#### Angle Measurements

**Figure 2:**
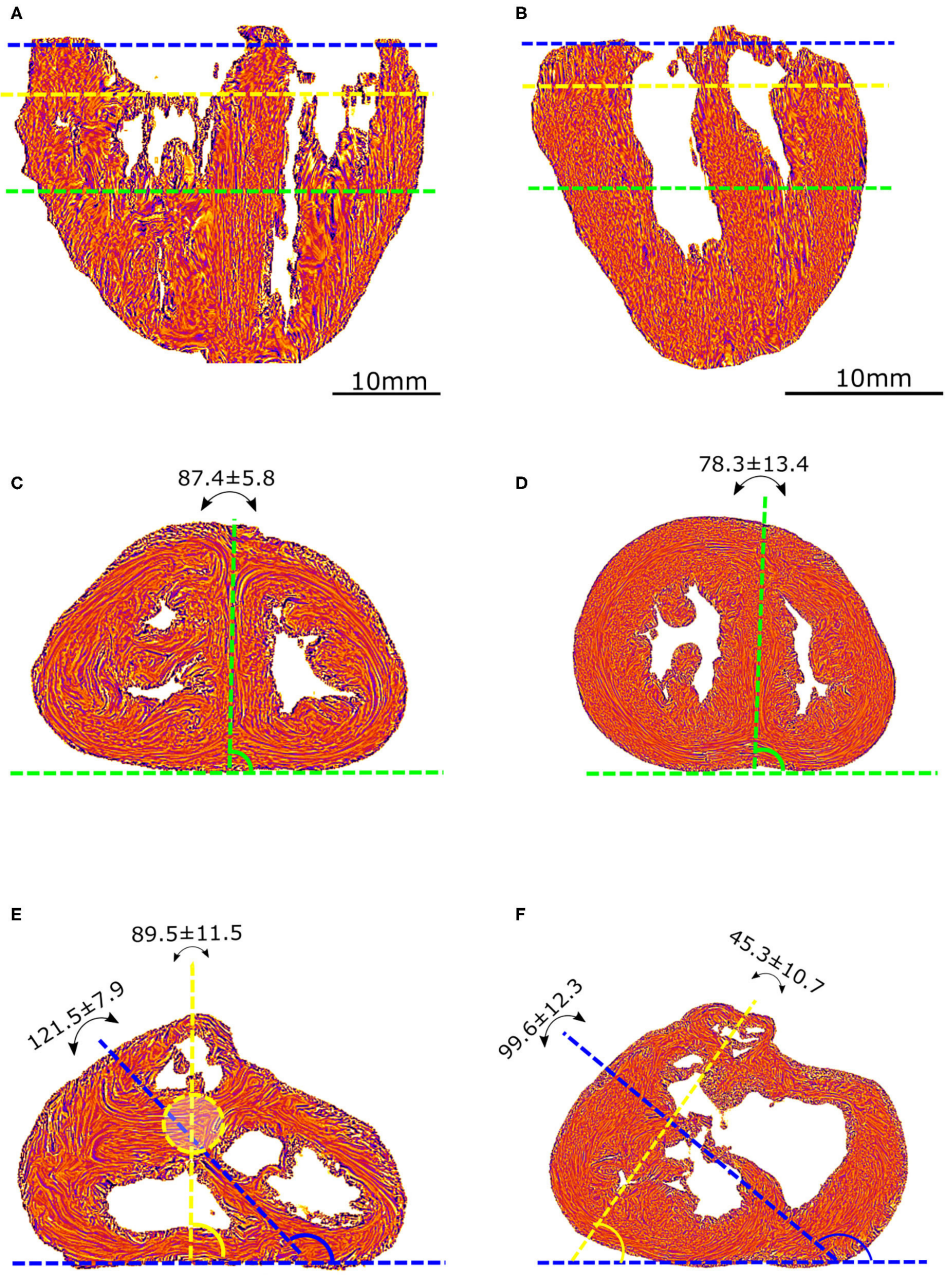
Morphometric measurements: left column-control, right column-ToF. **(A,B)** Long axis view, the three dotted lines show the different levels along the ventricular mass that are imaged in **(C–F)**: equatorial (green), aortic (yellow), and pulmonary valves (blue). **(C,D)** Short axis view equatorial level: IVS-eq angle (green dotted lines, average angle in degrees +/– standard deviation). **(E,F)** short axis view atrio-ventricular level: IVS-AV angle (blue dotted lines), Ao-PA angle (yellow dotted lines). The yellow dotted circle in **(E)** represents the projection of the aortic valve plan at the level of the pulmonary plan. At the apical level, no difference is measured between the angles in the control and ToF groups (*p* > 0.05). At the basal level, a statistically significant difference was measured between the control and ToF group for the IVS-AV angle and the Ao-PA angle (*p* < 0.01).

Angles were measured on standard short-axis sections, following trigonometric convention and with the 0° azimuth line defined as the inferior border of the inferior ventricular wall (Figure 2).

The interventricular septum angle at the biventricular mass equator level (IVS-eq) is defined as the angle from the 0° line to the axis of the latitudinal alignment of the cardiomyocytes of the interventricular septum (IVS).

The interventricular angle at the atrioventricular junction level (IVS-AV) is defined as the angle from the 0° line to the axis of latitudinal alignment of the cardiomyocytes of the IVS.

The Ao-PA angle at the atrioventricular junction level is defined as the angle from the 0° line to the line passing through the midpoints of the aorta (Ao) and pulmonary artery (PA) rings. In the case of pulmonary atresia, the line passes through the midpoints of the remnant of the muscular floor situated between the blind outflow tract of the right ventricle and pulmonary trunk.

## Results

A statistically significant difference (*p* < 0.01) was measured between the Abl values of the control and ToF group, see Table 1. Concerning the angles, although no statistically significant difference was measured between the IVS-eq angles of the control and ToF group, a statistically significant difference (*p* < 0.001) was measured between both the IVS-AV and the Ao-PA angles of the control and ToF group (Table 1). No correlation was measured between the Abl values and any of the three angle measurements in the control group or in the ToF group.

**Table 1:** Morphometric measurement (mean ± standard deviation).

	**Abl**	**IVS-eq(^**°**^)**	**IVS-AV(^**°**^)**	**Ao-PA(^**°**^)**
Control group	3.35 ± 0.46	87.4 ± 5.8	121.6 ± 7.9	89.9 ± 11.6
ToF group	2.04 ± 0.50	78.3 ± 12.3	99.7 ± 12.3	45.3 ± 10.7
*p* values	<0.01	>0.05	<0.001	<0.001

### Virtual Dissection of the Ventricular Mass

**Figure 3:**
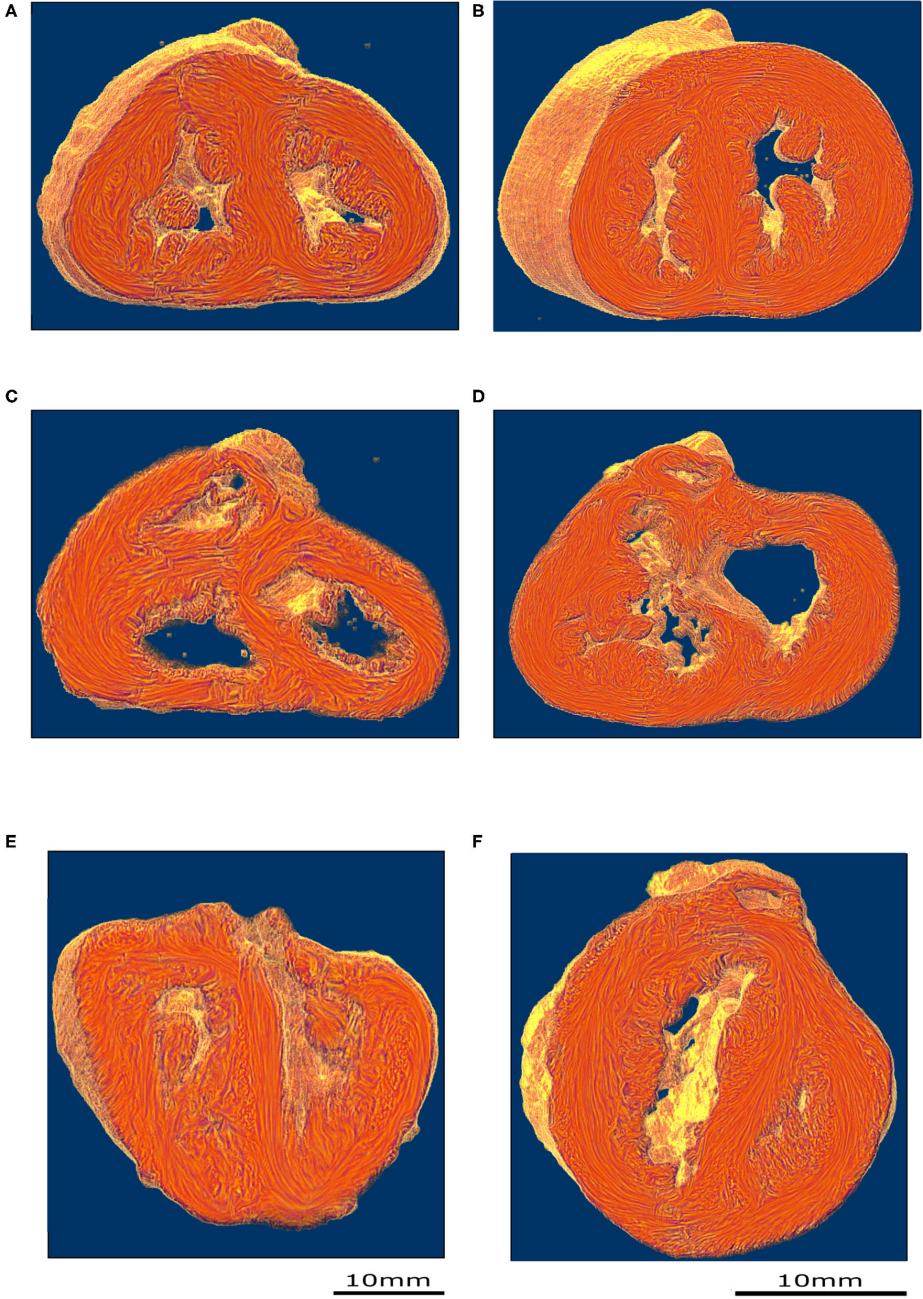
Virtual dissection of the ventricular mass LIC maps: left column-control, right column-ToF. Upper row, equatorial level **(A,B)**; middle row, basal level **(C,D)**; lower row **(E,F)** oblique sections. From the equatorial level to the apex **(A,B)**, no marked difference in the 3D architecture of the myocardial cells was found between the two groups. At the basal level, in the control hearts, the deep muscular continuity of the VIF and the IVS is verified **(C)**. In short axis section, the deep alignment of the cardiomyocytes of the VIF connect to the RVW while diverging as a Japanese fan. At its other end, the cardiomyocytes of the VIF connect to the IVS while diverging in two parts, one upper toward the OS and one lower toward the inferior part of the IVS. An oblique view **(E)** sharpens the description of the VIF and the IVS and their relationship with the septal trabeculations. At the basal level, in the ToF hearts, the muscular attachment of the VIF to the RVW remains. The other end of the VIF points to the lower part of the IVS positioned below the interventricular communication **(D)**. Concerning the OS, the deep latitudinal alignment of the cardiomyocytes bonds together the left part of the OS to the anterior wall of the left ventricle. The right end of the muscular OS bonds directly to the RVW superior to the attachment of the VIF. The right side of the OS makes a new muscular attachment directly to the RVW superior to the VIF **(E)**.

PLI reveals the deep cardiomyocyte alignment running in the ventricular walls and the IVS between more obliquely inclined sub-epicardial and sub-endocardial cardiomyocytes (see Figures 3A,B and Supplementary Material). There is no sharp transition in the progressive variation of orientation of cardiomyocyte alignment inside the ventricular walls and no boundaries visible within the heart.

In the apical half of the ventricular mass, from the equatorial level to the apex, although the internal cavities could have different shapes, star-shaped in the control group, crenellated crescent-shaped in the ToF group, no marked difference in the 3D architecture of the myocardial cells was found between the groups. The main differences were found in the basal half of the ventricular mass, from the equatorial level to the atrioventricular orifices and the right ventricular outflow tract (RVOT).

**Figure 4:**
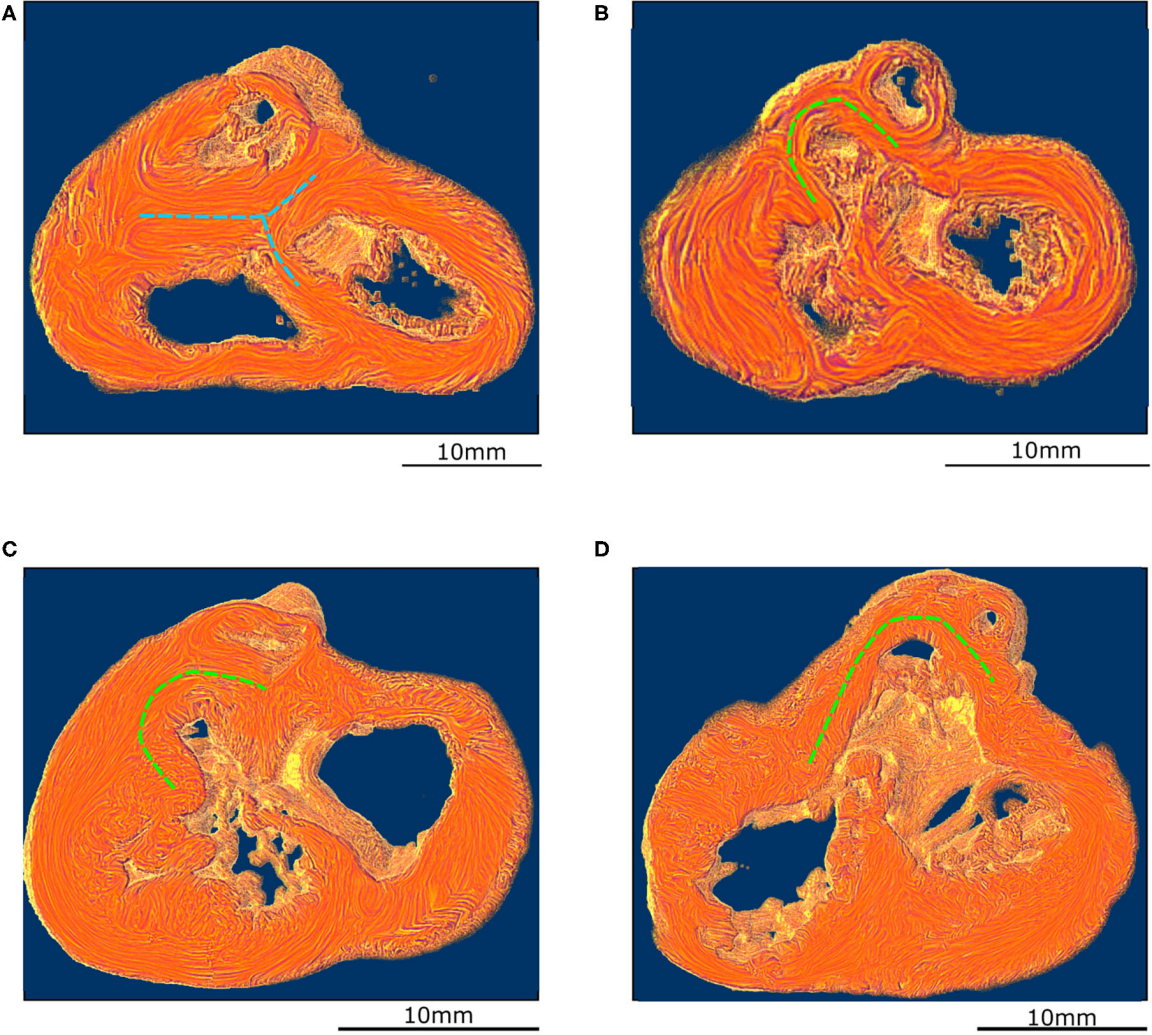
Virtual dissection of the ventricular mass to highlight the variability of the VIF. **(A)** Control heart–normal pattern, (blue dashed lines), **(B)** regular ToF, **(C)** ToF with severe hypoplasia of the outflow tract and **(D)** ToF with atretic pulmonary valve. The protrusion of the VIF in the right ventricular cavity is also variable. These three structures: the VIF, the RVW, and the OS define a muscular arch overriding the interventricular communication and the outflow tract of the left ventricle as an “inverted U,” well-seen in the short axis section **(D,B)**. This “inverted U” muscular arch (green dashed lines) is present in all observed ToF of this study, irrespective of the size of the different constitutive parts of the RVOT.

In all the specimens of the control group, there is a deep muscular continuity between the right ventricular wall (RVW), the ventriculo-infundibular fold (VIF), and the IVS (see Figures 3C, 4A and Supplementary Video 1). Viewed in the short-axis section, the deep alignment of the cardiomyocytes of the VIF connect to the RVW while diverging as a Japanese fan, whereas they connect to the IVS while diverging in two parts, one upper toward the outlet septum (OS) and one lower toward the inferior part of the IVS. An oblique view (Figure 3E) illustrates the description of the VIF and the IVS and their relationship with the septal trabeculations.

In all the specimen of the control group there was a free standing infundibular sleeve. In all the specimens of the ToF group, the muscular attachment of the VIF to the RVW remains. Though the other end of the VIF does not reach the IVS, it points toward where it is positioned below the ventricular septal defect (VSD) (see Figure 3D). The latitudinal alignment of the cardiomyocytes of the VIF and those of the upper part of the IVS, although separated, remain almost co-axial.

Concerning the OS, for all ToF specimens, the deep muscular attachment of its left part is constantly in continuity with the latitudinal alignment of the cardiomyocytes of the anterior wall of the left ventricle. The right side of the muscular OS makes a new muscular attachment directly to the RVW superiorly to the attachment of the VIF (Figure 3).

In ToF specimens with a pulmonary stenosis there was a free standing infundibular sleeve, reduced in size.

These three structures: the VIF, the RVW, and the OS define an “inverted U” that roofs the cone of space between the interventricular communication and the overriding aorta (Figures 3D, 4B). This “inverted U” muscular arch is present in all specimens of the ToF group included in this study, irrespective of the size of the different constitutive parts of the RVOT. However, the opening angle and the length of the branches of this “inverted U” largely depend on the size of the VIF, the distance between the points of insertion of the VIF and the OS into the RVW and the size of the OS that are highly variable (see Figure 4 and Supplementary Videos 2–4).

## Discussion

Our study concerned exclusively perinatal hearts and this must be considered when comparing with post-natal studies. The perinatal period was chosen for both theoretical and practical reasons. Theoretically, the period gives an insight into cardiac malformations after the completion of the embryonic period but before post-natal remodeling which differs between ToF and control hearts. Practically, the heart collection comes from a hospital perinatal pathology unit and the PLI methods developed for this study are well–suited to the size of perinatal hearts. The drawbacks are that perinatal hearts are rare and it is difficult to pair for size with control hearts. Indeed, there is a statistically significant difference in size between the control and ToF groups. We have, however, verified the independence of the angle measurements with the length measurements (Abl) in our collection. It is also noteworthy that the angle measurements fall within the post-natal range.

PLI enables the clear identification of the myoarchitecture of the different components of the right ventricle (the ventricular walls, VIF, OS, and IVS) both in the control and ToF groups. In the perinatal hearts of the control group, PLI shows a fairly simple deep myoarchitectural pattern with the muscular arch constituted by the muscular continuity between the RVW, the VIF and the IVS. PLI also sharpens the description of the deep bond (created by the deep cardiomyocyte alignment) modality of the VIF. Particularly noteworthy is the bimodal deep bond of the septal end of the VIF that diverges in two parts, one lower toward the inferior part of the IVS and one upper toward the OS. This muscular OS was clearly identified in each of the 10 control perinatal hearts studied. This is a difference compared with post-natal hearts, where the OS is, relative to the size of the RVOT, so small as to be sometimes almost virtual (13, 20, 28–30). The bridging position of the VIF in the hearts of the control group between the RVW and the IVS constitutes a muscular arch that plays a role in the functional coupling of the two ventricles: the classical “éperon” de Wolf (31, 32).

In the 10 perinatal hearts of the ToF group, the disconnection of the VIF and IVS disrupted this muscular arch. Virtual dissection of the ventricular mass clearly highlights the classical antero-cephalad deviation of the OS producing a subpulmonary infundibular obstruction, but also shows the deep bonds of the OS in the wall of the right and left ventricles. The OS bridges the anterior part of the right and left lateral ventricular walls, and constitutes a muscular arch whose role in the functional coupling of the two ventricles has still to be determined in the perinatal period (33). The arrangement of the OS with the RVW and the VIF forms an “inverted U” that roofs the cone between the interventricular communication and the overriding aorta. Each of the components of this “inverted U” is variable in form and size and is involved in the diversity of anatomical presentations of TOF in the perinatal period.

Although there are many articles devoted to the gross anatomy of ToF, to the best of our knowledge, only one study, Sánchez-Quintana, focuses on the 3D architecture of myocardial cells in ToF, in which post-natal hearts were manually dissected (10). Indeed, Sánchez-Quintana suggests the necessity to assess the angular changes through the whole ventricular mass; this has been achieved within our study by PLI and in previous studies by DTI-MRI. Despite the difficulty of comparing such different methods, it is possible to say that their description of the myoarchitecture of post-natal hearts with ToF is compatible with ours, although our technique allows to go further particularly in the fine description of the relationships of the VIF, the ventricular wall and the different components of the IVS.

As well as Sanchez-Quintana, we never observed boundaries separating discrete components inside the ventricular mass.

This absence of visible boundaries within the heart implies the incompatibility of our data with Torrent-Guasp's Model (34–42).

## Conclusion

The myoarchitecture of the ventricular mass is a new field of investigation allowed by the technological development of CT, Micro CT, PLI (for perinatal hearts), and DTI-MRI (for infant and adult hearts) that should be extended to the description of all cardiac malformations.

We applied PLI to the study of 10 perinatal hearts with ToF, compared with 10 control perinatal hearts to investigate the deep alignment of cardiomyocytes that bond the different components of the ventricular outflow tracts both together and to the rest of the ventricular mass.

For all ToF specimens studied, the deep latitudinal alignment of the cardiomyocytes bonds together the left end of the OS to the anterior wall of the left ventricle. And the right end of the muscular OS bonds directly to the RVW superior to the attachment of the VIF. Thus, the OS is a bridge between the lateral RVW and the anterior left ventricular wall. The VIF, RVW, and OS define an “inverted U” that roofs the cone between the interventricular communication and the overriding aorta. This “inverted U” muscular arch is present in all observed ToF specimens, irrespective of the size of the different constitutive parts of the RVOT. But the opening angle and the length of the branches of this “inverted U” depend on three components (the size of the OS, the size of the VIF, and the distance between the two points of their insertion into the RVW). The variation of these three components accounts for a large part of the diversity observed in the anatomical presentations of ToF in the perinatal period.

## Data Availability Statement

The datasets generated for this study are available on request to the corresponding author.

## Ethics Statement

The studies involving human participants were reviewed and approved by the institutional review board of Grenoble University Hospital. Written informed consent to participate in this study was provided by the participants' legal guardian/next of kin.

## Author Contributions

BT and GM: acquis datas. BT, P-SJ, and YU: the analysis of the results and to the writing of the manuscript. BT and P-SJ: wrote manuscript with support GM, JA, and YU. All authors contributed to the article and approved the submitted version.

## Conflict of Interest

The authors declare that the research was conducted in the absence of any commercial or financial relationships that could be construed as a potential conflict of interest.

## Supplementary Material

Supplementary Video 1 Video sequence through the reconstructed texture maps of a control perinatal heart.

Supplementary Video 2 Video sequence through the reconstructed texture maps of a perinatal heart with a regular form of ToF.

Supplementary Video 3 Video sequence through the reconstructed texture maps of a perinatal heart with ToF and a severe hypoplasia of the outflow tract.

Supplementary Video 4 Video sequence through the reconstructed texture maps of a perinatal heart with TOF with pulmonary atresia.
